# Delayed Intraluminal Hemorrhage Following Stapled Anastomosis in Abdominal Gunshot Trauma: A Case Report

**DOI:** 10.1002/ccr3.70674

**Published:** 2025-08-17

**Authors:** Daniel Scheese, Raisa Foushee, Emily Krueger, Jonathan Bennett

**Affiliations:** ^1^ Department of Acute Care Surgery Virginia Commonwealth University Richmond Virginia USA

**Keywords:** handsewn anastomosis, intestinal anastomosis, stapled anastomosis, surgery, trauma

## Abstract

Delayed intraluminal bleeding following stapled small bowel anastomosis, though rare, can result from trauma‐induced coagulopathy, systemic inflammation, and technical factors like staple load selection or clamping time. In trauma, intestinal anastomosis technique should be tailored to tissue condition, with handsewn anastomosis considered when appropriate.

## 1 Introduction

Intestinal anastomosis is a cornerstone of gastrointestinal surgery, restoring continuity following resection of the esophagus, stomach, small intestine, or colon. Over a century ago, the advent of intestinal staplers revolutionized surgical techniques, offering an alternative to traditional handsewn anastomoses [1]. Despite extensive research, including a Cochrane review by Neutzling et al., no definitive evidence establishes the superiority of either stapled or handsewn techniques in terms of safety or efficacy across different segments of the gastrointestinal (GI) tract [2, 3, 4].

Stapling is valued for its efficiency, reduced operative time, and minimal tissue manipulation, while handsewn techniques are often preferred for their perceived greater strength and reduced tendency to stricture [5]. Among anastomotic complications, leaks are the most feared due to their high morbidity and mortality. Studies emphasize tension‐free anastomoses between well‐perfused tissues as a strategy to reduce leak rates, without favoring either technique, including during emergency laparotomy [3] or in trauma patients with GI injury [6].

Conversely, anastomotic bleeding, though less studied, poses a significant challenge. While minor bleeding is usually self‐limiting, severe hemorrhage can occur, occasionally necessitating surgical intervention. Here, we describe a rare case of delayed intraluminal hemorrhage on postoperative day (POD) 2 following a stapled small bowel anastomosis in a patient following penetrating trauma, necessitating revision with a handsewn technique.

## 2 Case History

A 44‐year‐old male with no significant medical history presented to the trauma bay of our Level 1 Trauma Center after sustaining penetrating ballistic injuries to the left lower abdomen and left posterior lower back. Primary survey revealed an intact airway, equal breath sounds bilaterally, 2+ femoral pulses, and Glasgow Coma Scale score of 15. Initial vital signs demonstrated a narrowed pulse pressure (126/106 mmHg) with a normal heart rate of 72 bpm. The patient was normothermic on arrival (T 36.5°C). Chest radiographs demonstrated no evidence of hemothorax or pneumothorax. There was no evidence of retained projectile on abdominal X‐ray. There was no external hemorrhage or evidence of long bone fractures.

## 3 Methods

Initial resuscitation included placement of two large‐bore IVs and a rapid transfusion protocol. The patient was taken emergently to the operating room for an exploratory laparotomy.

### 3.1 Initial Operative Findings and Management

Intraoperatively, multiple small bowel enterotomies were identified in a focal segment of the jejunum, approximately 30 cm distal to the ligament of Treitz. Additionally, there was a penetrating injury to the descending colon mesentery, though no evidence of colonic injury or ischemia was observed. The injured segment of jejunum was resected using a GIA stapler with blue loads, and a stapled small bowel–small bowel anastomosis was performed with an additional blue load. The common enterotomy was closed with a 3–0 Vicryl suture in a Connell fashion and reinforced with 3–0 silk Lembert sutures.

Hemostasis was achieved at the site of the mesenteric injury, and the colonic mesenteric defect was closed with 3–0 silk sutures. To facilitate a planned second‐look procedure, an ABThera wound vacuum‐assisted closure device was applied. Following the surgery, a CT angiogram of the abdomen and pelvis, as well as a CT urogram, was performed to rule out missed injuries, such as ureteral damage, given the trajectory of the ballistic injury through the mesentery of the descending colon. These studies demonstrated no evidence of renal, ureteral, urinary bladder, or vascular injury within the abdomen or pelvis. However, findings confirmed postoperative changes, including partial small bowel resection with enteroenteric anastomosis and packing material in place.

### 3.2 Second‐Look Procedure

On POD 1, the patient underwent a planned return to the operating room. The descending colon appeared viable upon inspection, and the mesenteric injury was hemostatic. Evaluation of the proximal small bowel anastomosis demonstrated an intact and healthy repair. The entire small bowel was decompressed, with no evidence of intraluminal hemorrhage. A nasogastric tube was placed, and the abdomen was closed in a standard fashion. The patient was transferred to the intensive care unit (ICU) for close monitoring. Initial postoperative laboratory studies revealed a hemoglobin level of 12.8 g/dL.

### 3.3 Complication and Reoperation

On POD 2 following the initial surgery, the patient experienced sudden tachycardia and a precipitous drop in hemoglobin to 4.9 g/dL, raising concerns for active hemorrhage and hemorrhagic shock. Laboratory evaluation also revealed new‐onset thrombocytopenia, with a platelet count of 75 × 10^9^/L. A thromboelastography (TEG) study was obtained and did not demonstrate any coagulopathy. Notably, the patient had been receiving venous thromboembolism prophylaxis with enoxaparin at a dose of 0.5 mg/kg administered twice daily. The massive transfusion protocol was activated, and the patient was emergently taken back to the operating room for a repeat exploratory laparotomy.

Upon reentry, the small bowel was markedly distended with approximately 1.5 L of coagulated blood throughout its lumen. The colonic mesentery and retroperitoneum showed no active bleeding. Examination of the stapled small bowel anastomosis revealed diffuse oozing along the staple line. The anastomosis was resected using a GIA stapler with blue staple loads.

### 3.4 Handsewn Anastomosis Revision

A new handsewn anastomosis was performed. After creating enterotomies in both bowel ends with cautery, the posterior layer was constructed using 3–0 Vicryl in a running locking pattern, and the anterior layer was closed with a Connell suture. The repair was reinforced with loose imbrication using 3–0 silk Lembert sutures. The abdomen was closed, and the patient was returned to the ICU in stable condition.

## 4 Conclusions and Results

The patient demonstrated a return of bowel function on POD 5 and tolerated advancement to a regular diet. By POD 7, he was discharged home in good condition. At his most recent follow‐up, the patient reported no complications related to the handsewn anastomosis and continued to recover uneventfully.

## 5 Discussion

This case highlights a rare but serious complication of a stapled small bowel anastomosis performed in the setting of penetrating abdominal trauma. The occurrence of a massive intraluminal hemorrhage from the staple line approximately 36 h after the procedure underscores the multifactorial risks associated with trauma surgery and raises critical considerations regarding stapling techniques and patient‐specific factors.

### 5.1 Clinical Significance of the Case

Stapled anastomoses are commonly performed in trauma surgery due to their efficiency and reliability. At our institution and in the broader literature, anastomotic bleeding following stapled repairs is infrequent. However, when it occurs, bleeding may result from improper alignment or inadvertent trapping of vascular structures within the staple line, leading to pulsatile or delayed hemorrhage [7]. While most bleeding from anastomoses occurs intraoperatively or immediately postoperatively, delayed intraluminal hemorrhage is less common, making this case particularly noteworthy.

### 5.2 Physiological Contributors to Delayed Hemorrhage

In trauma patients, delayed anastomotic bleeding can result from complex physiological mechanisms, including trauma‐induced coagulopathy (TIC). TIC is a common condition in severely injured patients, characterized by a dysregulated coagulation cascade driven by significant tissue injury and hypoperfusion. Early‐phase TIC involves hyperactivation of anticoagulant pathways, including the protein C system, leading to degradation of clotting factors and impaired clot formation [8, 9, 10]. Additionally, platelet dysfunction and fibrinogen consumption further compromise hemostatic stability [11, 12, 13].

As the patient's condition evolves, TIC may transition to a procoagulant or hyperfibrinolytic state, destabilizing clot formation [14, 15]. This process can be exacerbated by repeated surgical interventions, endothelial damage, and the systemic inflammatory response. Endothelial glycocalyx shedding, a consequence of trauma and subsequent surgeries, disrupts vascular integrity and promotes hyperfibrinolysis, platelet dysfunction, and inflammation, increasing the risk of delayed hemorrhage [16, 17, 18].

Management of such patients must extend beyond surgical technique to include comprehensive physiological resuscitation. This includes early recognition and reversal of coagulopathy through goal‐directed therapy guided by viscoelastic testing modalities such as TEG or rotational thromboelastometry (ROTEM). In this case, the need for a second‐look laparotomy may have amplified systemic inflammation, further activating TIC and contributing to the hemorrhagic event.

### 5.3 Technical Factors in Stapled Anastomoses

Technical considerations in stapled anastomoses are critical to minimizing complications like bleeding or leaks. The type of stapler cartridge, clamping duration, and potential use of reinforcement materials all play a role in determining the integrity of the staple line. In this case, a GIA stapler with a blue cartridge (3.8 mm staple height) was used. This cartridge is typically appropriate for medium‐thickness tissues, such as the small bowel or colon. However, an increasing number of surgeons report using a white cartridge (2.5 mm staple height) for small bowel anastomosis to achieve a tighter closure, thereby reducing the risk of bleeding by minimizing vessel perforation. Conversely, using a staple load too tall for the tissue may result in incomplete compression, leaving gaps that predispose to bleeding or leaks [19, 20].

The technical decision to use a stapler must consider bowel wall edema, tissue thickness, and perfusion. Staplers, while fast and reproducible, were not originally designed for the inflamed, edematous, or friable bowel often seen in trauma. Although literature is limited, handsewn anastomosis may be advantageous in trauma due to its adaptability to tissue conditions and elimination of staple‐related risks. Our case does not suggest superiority of handsewn over stapled techniques broadly, but rather highlights the importance of tailoring the technique to the intraoperative scenario.

### 5.4 Staple Line Reinforcement and Clamping Time

Reinforcing the staple line with synthetic or biologic materials has been shown to reduce bleeding and leaks in gastrointestinal surgeries, particularly in tissues prone to fragility or edema [21, 22, 23]. While not routinely used in small bowel anastomoses, reinforcement may offer benefits in trauma or inflammatory settings where tissue integrity is compromised.

Additionally, the duration of stapler clamping before firing is an important but often overlooked factor. Pre‐compression of the tissue for 15–30 s before firing can help express edema, ensure uniform tissue thickness, and improve staple formation. Studies have shown that this practice reduces bleeding complications and promotes a more secure closure [24, 25, 26]. In contrast, shorter clamping times may lead to inadequate compression and an increased risk of complications.

### 5.5 Comparison With Handsewn Techniques

Handsewn anastomoses eliminate the risks associated with stapler malfunction, misalignment, or vascular entrapment. While more time‐intensive, they may be particularly beneficial in cases where tissue fragility, inflammation, or other risk factors increase the likelihood of complications. In this case, the successful revision with a handsewn technique underscores its utility in certain high‐risk scenarios.

## 6 Conclusion

This case underscores the dynamic and multifactorial nature of delayed hemorrhage following stapled small bowel anastomosis. Trauma‐induced coagulopathy, systemic inflammation from repeated surgical interventions, and technical factors related to the stapling process likely contributed to the hemorrhagic complication.

To mitigate such risks, surgeons must employ meticulous stapling techniques, select appropriate staple loads, allow adequate clamping time before firing, and consider the judicious use of handsewn anastomoses in select cases. Awareness of both common and rare complications is essential for optimizing outcomes in trauma patients, emphasizing the importance of tailoring surgical approaches to each patient's unique clinical context.

## Author Contributions

**Daniel Scheese:** conceptualization, investigation, supervision, writing – original draft, writing – review and editing. **Raisa Foushee:** conceptualization, writing – original draft, writing – review and editing. **Emily Krueger:** writing – original draft, writing – review and editing. **Jonathan Bennett:** supervision, writing – review and editing.

## Disclosure

The authors have nothing to report.

## Consent

Written patient consent was obtained prior to submission of the case report.

## Conflicts of Interest

The authors declare no conflicts of interest.

## Data Availability

Data sharing not applicable to this article as no datasets were generated or analyzed during the current study.
